# Pain without presence: a narrative review of the pathophysiological landscape of phantom limb pain

**DOI:** 10.3389/fpain.2025.1419762

**Published:** 2025-02-18

**Authors:** Hong Wu, Chandan Saini, Roi Medina, Sharon L. Hsieh, Aria Meshkati, Kerry Sung

**Affiliations:** ^1^ Department of Physical Medicine and Rehabilitation, Rush University Medical Center, Chicago, IL, United States; ^2^ Department of Physical Medicine and Rehabilitation, Emory University School of Medicine, Atlanta, GA, United States; ^3^ Rush University Medical College, Chicago, IL, United States

**Keywords:** amputation, phantom limb pain, pathophysiology, genetics, central nervous system, peripheral nervous system, psychology

## Abstract

Phantom limb pain (PLP) is defined as the perception of pain in a limb that has been amputated. In the United States, approximately 30,000–40,000 amputations are performed annually with an estimated 2.3 million people living with amputations. The prevalence of PLP among amputees is approximately 64%. Over the years, various theories regarding the etiology of PLP have been proposed, with some gaining more prominence than others. Yet, there is a lack of consensus on PLP mechanisms as the current literature exploring the pathophysiology of PLP is multifactorial, involving complex interactions between the central and peripheral nervous systems, psychosocial factors, and genetic influences. This review seeks to enhance the understanding of PLP by exploring its multifaceted pathophysiology, including genetic predispositions. We highlight historical aspects of pain theories and PLP, examining how these theories have expanded to include psychosocial dimensions associated with chronic pain in amputees. Additionally, we present significant findings from both human and animal studies focused on neuroaxial systems and recent advances in molecular research to further elucidate the complex and multifactorial nature of PLP. Ultimately, we hope that the integration of current theoretical frameworks and findings will lay a more robust foundation for future research on PLP.

## 1 Introduction

Phantom limb pain (PLP) is defined as the pain in a limb that has been amputated, but it can also occur following the loss of other body parts, such as an eye, breast, or tooth (1, 2). This phenomenon was first documented by the French barber-surgeon Ambroise Paré in 1551, who observed the condition in soldiers who had undergone battlefield amputations (3, 4). In 1797, British Admiral Horatio Nelson, after losing an arm in a battle, described the vivid sensation of his missing arm and the transient pain in his stump (5, 6). It was not until the 19th century, during the American Civil War, that neurologist Silas Weir Mitchell contributed significantly to the understanding of the PLP (7, 8).

In the United States, the annual incidence of limb amputations is approximately 464,644 (9), and the global prevalence of PLP among amputees is estimated at 64% (10). Projections indicate that by 2050, over 3.6 million individuals may be living with limb loss (11). PLP is not only physically debilitating but also profoundly impacts mental health, often leading to decreased quality of life, depression, and anxiety (12–14).

Despite extensive research, the origin of PLP remains elusive. Several mechanisms have been proposed to explain its development, with early theories focusing on religious, psychiatric, and psychological interpretations (15, 16). Subsequently, neurobiological and psycho-cognitive models emerged, suggesting a complex interplay of both psychological and physiological factors. Early studies primarily attributed the cause of PLP to the peripheral nervous system (PNS), particularly based on the discovery of ectopic discharges originating from the neuroma at the stump (17, 18). Later research identified the involvement of the central nervous system (CNS) in PLP, highlighting the role of maladaptive cortical plasticity in PLP's development (19–21). More recent advances point to multifactorial pathophysiology, with PLP likely arising from the convergence of multiple interconnected mechanisms (2, 22, 23).

Although previous reviews have addressed the contributions of the PNS, CNS, and psychological mechanisms to the development of PLP (24–28), and have offered historical perspectives (28, 29), our review aims to provide an overview of both pathophysiological and historical insights. We focus on important human and animal studies across neuroaxial systems and the latest advancements in molecular, psychosocial, and genetic factors—areas that have been less thoroughly explored in previous literature. Furthermore, we examine other predisposing risk factors that influence the development and persistence of PLP.

### 1.1 Peripheral nervous system

Early models of PLP proposed that abnormal firing of sensory nerves in the stump led to misinterpretation by the brain as sensations from the missing limb. PLP was thought to arise from disrupted sensory input after amputation, with peripheral nerve mechanisms playing a central role (30–32). The discovery that neuromas—abnormal nerve tissue growths—generate ectopic discharges further supported the idea that the stump may be a major source of pain. Ectopic activity, characterized by spontaneous neuronal firing, was observed in neuromas at the amputation site and appeared to contribute to PLP (33–41).

### 1.2 Nerve damage and neuroma formation

After amputation, nerve fibers at the distal end undergo retrograde degeneration, while nerve fibers at the proximal end undergo sprouting to elongate to reconnect to the distal end (42, 43). During the sprouting process, axon regeneration may occur in an unstructured manner leading to neuroma formation at the amputation site (44, 45). Histopathological studies using light and electron microscopy have extensively examined neuroma formation (46–49). During the early weeks post-injury, most axons terminate in smooth, elliptical swellings known as “neuroma end bulbs,” with minimal regenerative sprouting. Interestingly, the period of greatest electrical hyperexcitability in the neuroma coincides with this minimal sprouting, suggesting that the end bulbs, rather than extensive axonal regrowth, are the primary source of abnormal impulse discharges associated with neuropathic pain (46, 48, 49).

Oliveira et al. observed a cascade of regenerative events following peripheral nerve injury, including neuronal sprouting and neuroma formation, with associated abnormal afferent activity (47). These neuromas exhibited increased ectopic activity and heightened sensitivity to mechanical stimuli, changes attributed to altered ion channel dynamics, such as upregulation of voltage-gated sodium channels (Na_v_) (50–53). In a similar study, Wall et al. induced neuroma formation in rats by sectioning the femoral nerve, finding that stimulation at the tip of the neuroma elicited a significant response, unlike stimulation at the proximal nerve or dorsal root ganglion (DRG). This response was attributed to fine nerve fibers within the neuroma that exhibited spontaneous activity without external stimuli, a phenomenon not seen in intact roots. These fibers were also sensitive to mechanical pressure and could be inhibited by lidocaine, further highlighting the neuroma's role in generating spontaneous, abnormal nerve impulses (54). These peripheral mechanisms, including ectopic activity, ion channel dysregulation, and disrupted sensory input, collectively contribute to the onset and persistence of PLP.

### 1.3 Sympathetic involvement

In addition to neuroma formation contributing to the development of PLP, the sympathetic nervous system (SNS) also plays a role in the development of PLP through several mechanisms including ephaptic transmission, activation of nociceptors and low-threshold mechanoreceptors, and sympathetic coupling in the periphery and DRG (55).

In animal studies, beta-adrenergic blockade leads to reduced sensation of PLP while adrenaline injections into neuromas lead to heightened sensation of pain and paresthesia, providing support for the sympathetic involvement in PLP (56–58). Sympathetic dysregulation in the stump of amputee patients has also been supported by evidence indicating that reduced surface blood-flow may be a physiologic correlate of the burning sensation in PLP (59). In addition, animal studies suggest increased postsynaptic norepinephrine release during emotionally stressful situations correlates with hyperalgesia and heightened spinal nociception. Changes in catecholamine levels and alpha-adrenoreceptor involvement have been implicated in the pain associated with neuroma formation, further underscoring the complexity of peripheral contributions to post-amputation pain syndromes (59, 60). Given the early onset of pain immediately after amputation and the inability of anesthetic blocks to eliminate PLP entirely, peripheral factors and the SNS alone cannot be considered the sole factors contributing to PLP but should be regarded as key factors.

### 1.4 Peripheral sensitization

Peripheral sensitization is a process where the peripheral nerves become more sensitive to stimuli following injury or inflammation and is believed to be influenced by the formation of neuromas. As mentioned above, neuromas can produce spontaneous ectopic discharges and lead to hyperexcitability and enhanced sensitivity to normally non-painful stimuli, a phenomenon known as allodynia (61, 62). Additionally, peripheral sensitization can result from the release of inflammatory mediators and neurotransmitters that further sensitize the nerves, creating a cycle of pain amplification (63–65). Although the exact mechanism for development and maintenance of chronic ectopic firing is not fully understood, Na_v_ has been shown to contribute towards increased ectopic firing (66–69).

Additionally, recent investigations into the molecular pathways following peripheral nerve injury have revealed the release of adenosine triphosphate (ATP), by primary sensory and dorsal horn neurons, that bind to P2X receptors expressed by microglia adjacent to the dorsal horn (DH) (70). The binding of ATP leads to the release of brain-derived neurotrophic factor (BDNF) into the DH, increasing neuronal hyperexcitability and nullifying the inhibitory responses, as discussed above. Another recent study using mice models has further delineated this understanding (71). In this study, wild-type mice were compared to mutant mice who had a deletion of a gene that codes for a microglia-specific ATP releasing channel. The examiners found that the mutant mice, who lacked the ATP-releasing channels, had reduced allodynia in comparison to the wild-type mice (71).

Several animal and human studies have demonstrated the role of Na_v_ in nociceptive sensation and expressivity/peripheral sensitization following peripheral nerve injury (72, 73). These findings suggest that the upregulation of the Na_v_ channels leads to increased nerve excitability that manifests as hyperalgesia. One potential mechanism for the accumulation of these receptors within neuromas is through membrane remodeling following axotomy (66, 67). Immunostaining of human neuroma tissue has demonstrated that individuals with neuropathic pain have increased expression of Na_v_ channels and ankyrin G, which is a protein involved in regulating Na_v_ (74).

In addition to molecular mechanisms involving the PNS in the development of PLP, there is evidence suggesting that re-innervation by motor neurons of residual proximal muscles contributes to abnormal nerve firing. Animal studies have demonstrated that motor neurons, which formerly innervated distal target muscles, survive and re-innervate new targets in residual muscles (75, 76). Sensory afferent neurons from both the residual stump and skin have been observed re-innervating territories in the cuneate nucleus (77, 78). Because the cuneate nucleus relays sensory information to the somatosensory cortex, its stimulation by stump muscles can lead to phantom limb sensations (PLS) and possibly PLP (77).

## 2 Central nervous system

As the complexity of PLP became more apparent, researchers began to integrate the CNS's involvement. It became clear that PLP was not solely explained by peripheral mechanisms or simple gate modulation. The concept of cortical reorganization emerged as a key factor in the development of PLP. Studies, including those by Flor et al., demonstrated that after amputation, the brain's somatosensory cortex, which once represented the missing limb, undergoes reorganization (79). The cortical areas that previously mapped the amputated limb may become “invaded” by adjacent body areas, such as the face or residual limb. This reorganization may lead to the perception of sensations or pain in the absent limb (i.e., phantom limb sensations). This maladaptive plasticity in the brain could underlie the chronic pain and sensory disturbances often experienced in PLP.

### 2.1 Cortical reorganization

The understanding of PLP has evolved significantly through two main theoretical frameworks: Melzack and Wall's gate control theory (80) and Melzack's later neuromatrix theory (81). Both emphasize the CNS' role in modulating pain. The gate control theory posited that pain is not simply the result of sensory input but involves modulation within the spinal cord and brain. The theory introduced the concept of “gates” in the spinal cord that can either inhibit or facilitate pain transmission to the brain. In PLP, these gates can become sensitized after amputation, allowing abnormal pain signals to reach the brain (80). Building on this, Melzack's “neuromatrix” theory proposed that pain arises from a “neurosignature,” produced by a genetically determined synaptic architecture (neuromatrix) in the CNS. This theory expanded pain perception to include sensory, emotional, and cognitive components, explaining how the loss of sensory input from the amputated limb leads to abnormal brain activity, which misinterprets this activity as pain or sensation in the missing limb (81).

The concept of maladaptive cortical reorganization has been widely debated as a key mechanism in the CNS origin of PLP (19, 21, 81–83). This process occurs in the primary somatosensory cortex (S1) and primary motor cortex (M1), where reduced sensory input, such as from limb amputation, leads to a decreased cortical representation of the amputated body part and the subsequent expansion of adjacent body parts' cortical representations (84–86). The phenomenon arises when distal axons of the DRG become disconnected from their targets following amputation, generating ectopic activity in the residual limb. This abnormal signaling within the spinothalamic tract triggers cortical reorganization, as neighboring cortical regions invade the deafferented areas. These changes can manifest as both non-painful and painful sensations, such as PLS and PLP, in the absence of peripheral input (81). Early animal studies, such as Rasmusson's work on raccoons, demonstrated that the loss of a digit led to cortical changes, with the sensory map corresponding to the missing digit being taken over by the neighboring cortical area, leading to heightened sensitivity (86). Kaas et al. (84) showed that sensory cortical maps in primates reorganized after injury (85). Similar results were obtained in adult monkeys, owls, and squirrels, where sensory maps underwent significant alterations in response to sensory loss (87–90).

Human studies further elucidated the role of cortical reorganization in PLP. In a landmark 1995 study, Flor et al. utilized magnetoencephalography (MEG) and magnetic resonance imaging (MRI) to demonstrate that cortical reorganization in upper-limb amputees was associated with shifts in the locus of cortical responsiveness, particularly in those experiencing PLP (79). Flor's later work on congenital limb absence revealed that individuals born without limbs exhibited minimal cortical reorganization and no PLP (21), suggesting that reorganization is critical for PLP development. Furthermore, advanced neuroimaging, particularly functional MRI (fMRI), has challenged the idea that cortical reorganization alone accounts for PLP (20, 91–93). Makin et al. found that despite preserved cortical maps in amputees with PLP, disrupted inter-regional connectivity may contribute to PLP (94). Similarly, Andoh et al. observed increased activation in motor and sensory cortices, but this activation was not correlated with PLP intensity, suggesting a multi-factorial nature of the condition (93).

However, this static model of the cortical reorganization theory did not fully account for the variable and sometimes reversible nature of PLP, such as fluctuation of PLP and changes of PLP responding to interventional treatments. These observations suggest that cortical changes are not static but can be modulated or dynamically reorganized. In the late 1990–2000s, the theory of dynamic cortical reorganization emerged, and refers to a continuous reshaping of the cortical maps in response to external stimuli, motor and sensory feedback, proposing that reorganization involves both structural and functional changes, particularly in how the brain processes sensory and pain signals. Flor et al. 1995 showed that the cortical changes in response to somatosensory evoked potentials (SEPs) were related to the intensity of PLP (79). The pioneering work on mirror therapy from Ramachandran et al. in 1996 (95) revealed that visual feedback can reduce PLP by alternating the cortical map in real-time. Schwenkreis et al. used transcranial magnetic stimulation (TMS) to show that the motor cortex reorganizes to incorporate adjacent body parts after amputation, supporting the notion of dynamic brain plasticity (96). Additionally, studies demonstrated that emotional and cognitive activities can also influence the brain' response to pain suggesting a role of cognitive-behavioral therapy (CBT) in treating PLP (55).

### 2.2 The thalamic contributions

Thalamus, a critical relay center for sensory and motor information, plays a key role in the CNS origin of PLP (97, 98). In a typical nervous system, primary afferent pain signals from peripheral nociceptors synapse at the DH of the spinal cord and ascend via the spinothalamic or spinoreticular tracts (79, 99–101). These signals pass through the brainstem, where facilitatory or inhibitory signals modulate pain transmission before reaching the thalamus for further processing (102–105). After amputation, the thalamus undergoes reorganization similar to cortical changes, with neurons initially responsible for the amputated limb's sensory processing now responding to inputs from neighboring body areas (78, 93, 106, 107). This leads to the misperception of pain in the missing limb. The thalamus can become sensitized through an increase in Na + channels in thalamic neurons, akin to peripheral sensitization (108, 109). Studies show that the thalamic representation of the residual limb is enlarged in amputees compared to individuals with intact limbs, and micro-stimulation of the thalamus in the absence of peripheral stimuli can evoke phantom sensations and PLP (78, 86, 110, 111). This central sensitization, coupled with altered thalamic representations, contributes to the chronic nature and intensity of PLP (89, 105, 109, 112).

Additionally, the thalamus plays a key role in modulating pain perception. Changes in thalamic activity and connectivity affect how pain is processed, contributing to phenomena such as allodynia (pain from non-painful stimuli) and hyperalgesia (amplified pain responses) (113–115). The thalamus interacts with cortical and subcortical regions, including the somatosensory cortex, anterior cingulate cortex, and insula, which are involved in the sensory and emotional components of pain (116, 117). Deep brain stimulation (DBS) and transcranial stimulation (TCS) targeting the thalamus have shown efficacy in reducing PLP and other neuropathic pain conditions (118–124). These treatments support the thalamus's involvement in PLP, and ongoing research into their mechanisms may reveal new neuromodulation strategies to alleviate PLP symptoms.

### 2.3 Centralization of pain and windup phenomenon

Centralization refers to the increased sensitivity and responsiveness of neurons within the CNS, dorsal horns, and primary afferent fibers (125, 126). As mentioned previously, the DRG becomes hyperexcitable with increases in Na_v_, leading to ectopic firings that cause pain in the absence of stimuli (51–54, 127, 128). The process of central sensitization also involves sensitized C-fibers, which release glutamate and interact with neuropeptides and N-methyl-D-aspartate (NMDA) receptors to amplify spinal cord responses (126, 129–132).

The “wind-up” phenomenon, characterized by frequency-dependent increases in spinal cord neuron excitability due to C-fiber stimulation, serves as a precursor to central sensitization. Repetitive stimulation during wind-up can lead to an expanded receptive field, a significant feature of central sensitization (133, 134). Wind-up differs from central sensitization in its temporal nature, ceasing after the stimulus ends, while central sensitization can persist (126, 133, 134). This process, critical in demonstrating spinal cord plasticity, amplifies pain signaling and sets the stage for chronic pain conditions such as PLP (126, 133, 134).

In amputees, increased nociceptive activity is attributed to the loss of descending inhibitory control, particularly through reduced gamma-aminobutyric acid (GABA) and glycine-mediated inhibition due to nerve injury (105, 135–137). This disinhibition occurs both in the spinal cord and cortex, as GABAergic interneurons are damaged by axotomy, contributing to spinal hyperexcitability (136–139). Additionally, brain-derived neurotrophic factor (BDNF) plays a role in post-injury neuroplasticity, promoting excitatory effects on nociception through NMDA receptor modulation (140–145). Animal studies have shown that spinal BDNF infusion enhances nociceptive responses, which can be mitigated by NMDA antagonists (143, 146). The interplay between disinhibition, BDNF, and NMDA receptors contributes to the complex mechanisms underlying PLP, underscoring the need for further investigation into these molecular pathways.

### 2.4 Proprioceptive memory

Proprioceptive memory, or the brain's ability to retain awareness of body position. Even after amputation, amputees often report sensations of proprioception in the missing limb (147, 148). One theory suggests that proprioceptive information is consolidated as long-term memory during repeated motor tasks, allowing these memories to persist despite the absence of the limb (148, 149). This theory is supported by studies where individuals could sense the position of their amputated limbs even after regional anesthesia (150, 151).

The connection between proprioceptive memory and PLP is further highlighted by the phenomenon of frozen phantom limbs, which often mimic the limb's position prior to amputation, suggesting that these proprioceptive imprints remain intact (152–154). This preservation could explain therapeutic interventions like mirror therapy (MT), which aim to align visual and proprioceptive inputs, potentially clearing mismatched proprioceptive memories and alleviating PLP (95, 155). Mirror therapy has been shown to reduce PLP by providing visual feedback that matches proprioceptive input, addressing the sensory conflict that contributes to pain (156).

Moreover, PLP may arise from a mismatch between visual and proprioceptive inputs. The brain integrates visual cues with tactile and proprioceptive sensations to create body ownership, and any discrepancy between these signals may lead to the experience of PLP (157–160). These findings emphasize the complex relationship between proprioceptive memory and PLP and suggest that therapies targeting this interaction could offer novel ways to manage chronic pain following amputation.

## 3 Psychological factors

PLP is not only influenced by mechanisms involving the CNS and PNS but also by significant psychosocial components. Emotional and cognitive factors can influence the dynamics of cortical reorganization involved in PLP (79, 156, 161), contributing to maladaptive cortical reorganization and increased pain perception.

### 3.1 Stress and PLP

Chronic stress is one of the key psychological factors implicated in the exacerbation of PLP. Stress can influence neuroplastic changes in the brain, particularly in areas related to sensory and motor processing. Lotze et al. (162) demonstrated that stress was associated with cortical reorganization, thereby contributing to the sensation of PLP. Additionally, higher stress levels were linked to more intense pain in PLP patients (163, 164), suggesting that stress may exacerbate cortical maladaptation, leading to persistent pain. Furthermore, several studies (165, 166) have shown that psychological stress could modulate pain perception, including phantom pain. Stress-induced activation of brain areas involved in emotional regulation and pain processing, such as the anterior cingulate cortex, may increase sensitivity to pain stimuli, contributing to the experience of PLP.

### 3.2 Depression and PLP

Depression is another significant psychological factor associated with the onset and intensity of PLP. Depressive symptoms often co-occur with PLP, and patients with depression tend to report more severe PLS (13). Larbig et al. (167) found that higher levels of depression were associated with more severe PLP, and the presence of depression appeared to increase sensitivity to pain. This finding suggests that depression could alter pain processing mechanisms, potentially through disruptions in brain structures that regulate pain, such as the periaqueductal gray and serotonergic pathways. Additionally, Ahmed et al. (168) demonstrated that depressed amputees were significantly more likely to experience chronic PLP. They linked depression to lower serotonin levels, which are known to play a crucial role in pain modulation, thus reinforcing the idea that mood disorders contribute to the persistence and intensity of PLP.

### 3.3 Anxiety and PLP

Anxiety, particularly post-traumatic anxiety, is another important factor that can exacerbate PLP. Ramachandran and Hirstein (148) proposed that anxiety and fear might amplify the perception of PLP through increased central nervous system sensitivity. Anxiety related to the loss of a limb or concerns about complications may heighten the brain's pain processing capacity, making PLP more intense. Further supporting this notion, Desmond et al. (169) found that anxiety was correlated with more frequent and intense PLP and suggested that anxiety could enhance pain perception by activating brain regions involved in both emotional processing and pain, such as the amygdala and somatosensory cortex, which may increase the salience of pain and contribute to its persistence.

### 3.4 Emotional and cognitive factors in PLP

Cognitive and emotional factors, such as catastrophizing and negative emotions, could contribute to central sensitization (170, 171)—a phenomenon where the central nervous system becomes hyper-responsive to pain signals. This increased sensitivity may make PLP more intense and persistent. Several studies (171–174) found that negative emotional states and cognitive distortions could amplify the perception of pain through increased central sensitization. Individuals with post-traumatic stress disorder (PTSD) often report higher levels of PLP through neurobiological changes, such as the sensitization of pain pathways and emotional dysregulation (13). Numerous studies (175–177) supported this notion by showing that psychological interventions targeting depression and anxiety significantly reduced the intensity of chronic pain. This suggests that the psychological well-being of patients is crucial in managing PLP, as mental health treatment may help alleviate both the emotional burden and the neuroplastic changes associated with PLP.

## 4 Genetic influences

Previous studies have established the genetic associations with chronic neuropathic pain syndromes, specifically identifying associations within genes coding for voltage-gated ion channels, calcium binding genes, and mitochondrial phosphate caries (178–181). Identification of genetic involvement in neuropathic pain suggests a possible role of genetic predisposition in PLP, which could significantly enhance the clinical management and treatment outcomes of this condition. Devor et al. 2005 demonstrated heritable traits for neuropathic pain in rodents with sciatic nerve ligations, where specific genetic loci on chromosome 15 were linked to pain sensitivity and neuroma-related pain (182). These findings highlight the role of genetic variation in modulating pain responses and provide a framework for understanding the genetic underpinnings of PLP. Human genetic research on PLP is still in its early stages but has shown promising results. Notably, studies by Nissenbaum et al. and Bortsov et al. identified polymorphisms in the *CACNG2* gene (calcium voltage-gated channel auxiliary subunit gamma 2) on chromosome 22, which regulates AMPA receptor trafficking (183, 184). Specific polymorphisms in *CACNG2* were associated with chronic neuropathic pain, such as postmastectomy pain, suggesting a potential link to PLP (184). This discovery marks one of the first genetic associations with PLP in humans, though further research is needed to confirm these findings.

While a direct link between genetics and PLP remains unestablished, the studies in this area hold promise and could significantly benefit future exploration into the genetic factors contributing to PLP. The exploration of epigenetic mechanisms, such as microRNA regulation, in chronic neuropathic pain also holds promise for PLP research (185–187). These epigenetic factors may offer new insights into how environmental factors and genetic predispositions interact to influence pain perception and could lead to novel therapeutic strategies targeting gene expression (185–187).

## 5 Conclusion

PLP remains one of the most challenging and poorly understood conditions in the field of amputation, primarily due to the absence of a comprehensive consensus on its underlying pathophysiology. The multifactorial nature of PLP has led to ongoing debate regarding the specific mechanisms involved in its onset. Insights from cerebral, spinal, and peripheral perspectives provide critical evidence, suggesting that PLP likely results from the complex interaction of these diverse systems, rather than from a single etiological cause. Beyond the neurobiological underpinnings, recent advancements in psychology and genetics have shed light on the multifaceted mechanisms contributing to PLP's pathogenesis, further highlighting the intricate nature of the disorder.

This review has aimed to synthesize these diverse lines of research, providing an integrative overview of the physiological, psychological, and genetic factors implicated in PLP. By examining key human and animal studies, we have highlighted recent progress in molecular, psychological, and genetic research that is reshaping our understanding of this condition. A multidimensional approach to PLP, integrating these findings, holds the promise of more personalized treatment strategies that address the diverse and individualized needs of patients. Ultimately, such an approach has the potential to improve treatment outcomes, enhance patient well-being, and drive innovations in both PLP research and therapeutic interventions in the future.
